# Cell Death via Lipid Peroxidation and Protein Aggregation Diseases

**DOI:** 10.3390/biology10050399

**Published:** 2021-05-04

**Authors:** Katsuya Iuchi, Tomoka Takai, Hisashi Hisatomi

**Affiliations:** Department of Materials and Life Science, Faculty of Science and Technology, Seikei University, 3-3-1 Kichijojikitamachi, Musashino-shi, Tokyo 180-8633, Japan; dm206115@cc.seikei.ac.jp (T.T.); hisatomi@st.seikei.ac.jp (H.H.)

**Keywords:** lipid peroxidation, cell signaling, protein aggregation, non-apoptotic cell death

## Abstract

**Simple Summary:**

It is essential for cellular homeostasis that biomolecules, such as DNA, proteins, and lipids, function properly. Disturbance of redox homeostasis produces aberrant biomolecules, including oxidized lipids and misfolded proteins, which increase in cells. Aberrant biomolecules are removed by excellent cellular clearance systems. However, when excess aberrant biomolecules remain in the cell, they disrupt organelle and cellular functions, leading to cell death. These aberrant molecules aggregate and cause apoptotic and non-apoptotic cell death, leading to various protein aggregation diseases. Thus, we investigated the cell-death cross-linking between lipid peroxidation and protein aggregation.

**Abstract:**

Lipid peroxidation of cellular membranes is a complicated cellular event, and it is both the cause and result of various diseases, such as ischemia-reperfusion injury, neurodegenerative diseases, and atherosclerosis. Lipid peroxidation causes non-apoptotic cell death, which is associated with cell fate determination: survival or cell death. During the radical chain reaction of lipid peroxidation, various oxidized lipid products accumulate in cells, followed by organelle dysfunction and the induction of non-apoptotic cell death. Highly reactive oxidized products from unsaturated fatty acids are detected under pathological conditions. Pathological protein aggregation is the general cause of these diseases. The cellular response to misfolded proteins is well-known as the unfolded protein response (UPR) and it is partially concomitant with the response to lipid peroxidation. Moreover, the association between protein aggregation and non-apoptotic cell death by lipid peroxidation is attracting attention. The link between lipid peroxidation and protein aggregation is a matter of concern in biomedical fields. Here, we focus on lethal protein aggregation in non-apoptotic cell death via lipid peroxidation. We reviewed the roles of protein aggregation in the initiation and execution of non-apoptotic cell death. We also considered the relationship between protein aggregation and oxidized lipid production. We provide an overview of non-apoptotic cell death with a focus on lipid peroxidation for therapeutic targeting during protein aggregation diseases.

## 1. Introduction

**Figure 1:**
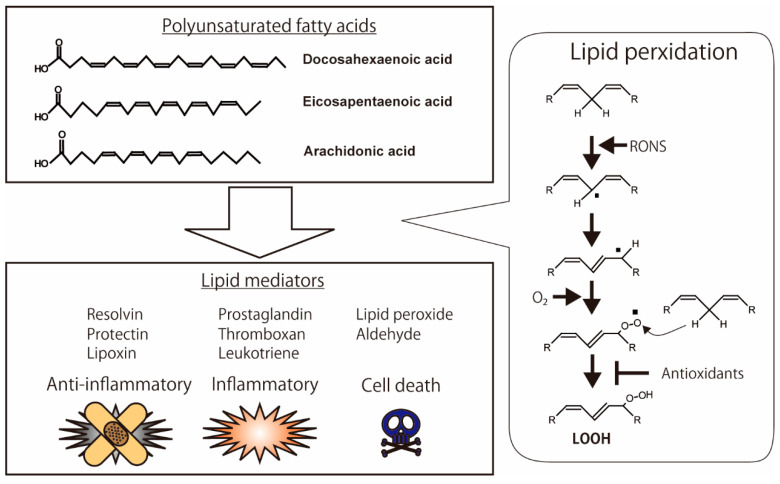
Oxidation of polyunsaturated fatty acids (PUFAs) and production of lipid mediators. Anti-inflammatory lipid mediators, such as resolvin, protectin, and lipoxin, are produced from PUFAs. Pro-inflammatory lipid mediators, such as prostaglandin, thromboxane, and leukotriene, are also metabolized from PUFAs. The highly reactive chemicals, including hydroperoxide and aldehyde, have the binding activities of biomolecules in the cells, which lead to cell death. The illustration was adapted from Iuchi (2021).

Lipids are essential for various biological functions. They play three important roles in cells: they maintain the cellular membrane structure, regulate cellular signaling, and store energy. Lipids protect cellular components. There are three types of membrane lipids: phospholipids, glycerophospholipids, and sphingolipids [1]. Plasma and organelle membranes are composed of lipids, which allow for the entry of selective substrates into cells or organelles. Saturated and unsaturated fatty acids are lipid components of triacylglycerides and phospholipids of the cellular membrane. Saturated fatty acids contain no double bonds in their fatty acids. On the other hand, unsaturated fatty acids have one or more double bonds in the fatty acid chains. Polyunsaturated fatty acids are oxidized by both enzymatic and non-enzymatic reactions. As shown in Figure 1, the polyunsaturated fatty acids containing bis-allylic methylene groups are sensitive to reactive oxygen and nitrogen species (RONS) (Figure 1). The non-enzymatic oxidation of unsaturated fatty acids is initiated by the abstraction of the hydrogen in the bis-allylic methylene groups by RONS. Next, the unsaturated fatty acids are oxidized, followed by the formation of alkoxyl and alkyl peroxyl radicals. These radicals attack other unsaturated fatty acids, leading to sequential oxidation, known as radical chain reactions [2].

Biomolecules such as DNA, proteins, and lipids in cells are directly oxidized by RONS under oxidative stress conditions, and they form oxidized DNA bases, misfolded proteins, and oxidized lipids, respectively. These oxidized biomolecules act as second messengers, which activate multiple cellular signaling pathways [3]. Cell death signaling pathways are activated by oxidized biomolecules. Protein aggregation diseases are associated with the dysfunction of tissues and organs following oxidative stress in the body [4]. The mechanisms underlying these diseases are involved in the dysfunction of organelles. The accumulation of aggregated proteins in the cytosol disturbs the organelle functions, oxidative phosphorylation in the mitochondria, protein synthesis in the endoplasmic reticulum (ER), and protein modification in the Golgi. Excess oxidative stress initiates the cell death signal. RONS directly damage proteins in the organelles. The precise mechanism underlying organelle dysfunction resulting from the aggregation of damaged biomolecules during cell death remains elusive. 

As well as the lipid peroxidation of the cellular membrane, the perturbation of intracellular iron homeostasis and redox state causes both apoptotic and non-apoptotic cell death concomitant with organelle dysfunction. The attenuation of lipid peroxidation is one of the targets for the prevention of neurodegenerative disorders [5]. The decrease in the thiols of peptides and proteins is associated with ferroptosis, which is a new type of non-apoptotic cell death via lipid peroxidation, as described below. Most recent reports have shown a relationship between neurodegeneration disease, which is associated with iron-dependent disease, and lipid peroxidation-induced cell death such as ferroptosis [6]. Lipid peroxidation products and protein aggregation concomitantly increase in neurodegenerative diseases. 

Protein misfolded oligomers are involved in the onset and progression of neurodegenerative disease. Oligomers are major targets for drug discovery in protein misfolding diseases [7] known to potentiate ROS production, and membrane composition is critical for the vulnerability of the cell to oligomers [8]. Targeting lipid peroxidation/RONS could, therefore, potentially prevent oligomerization and/or protect the cell membrane. 

Taken together, lipid-peroxidation-induced cell death is associated with protein aggregation, and leads to the disturbance of organelle and cellular functions. The mechanism by which lipid peroxidation induces cellular dysfunction in various diseases is pivotal, and its analysis is an emerging area of interest. This review summarizes the current views on the relationship between lipid peroxidation and protein aggregation in neurodegenerative diseases, such as Alzheimer’s disease (AD), amyotrophic lateral sclerosis (ALS), and Parkinson’s disease (PD). 

## 2. Lipid Peroxidation

Lipid peroxidation of cellular membranes is a radical chain reaction initiated by hydrogen abstraction in polyunsaturated fatty acids (PUFAs) with bis-allylic methylene. Highly reactive species of RONS, such as hydroxyl radicals, are essential for the abstraction of hydrogen [9]. Excess oxidative stress, under conditions of increasing RONS, causes lipid peroxidation of the cellular membrane, which leads to the intracellular accumulation of highly reactive products such as lipid peroxide (LOOH), malondialdehyde (MDA), hydroxynonenal (HNE), and acrolein; these products modify protein structure and function [10]. Highly unsaturated fatty acids, such as docosahexaenoic acid (DHA), eicosapentaenoic acid (EPA), and arachidonic acid (ARA), are sensitive to RONS. These oxidative products demonstrate anti-inflammatory, pro-inflammatory, and pro-cell death activities (Figure 1) [11]. Resolvin, protectin, and lipoxin in particular exhibit anti-inflammatory activities [12]. In contrast, prostaglandins, thromboxanes, and leukotrienes are inflammatory lipid mediators [13,14]. Antioxidants modulate lipid peroxidation by directly scavenging RONS and indirectly upregulating and downregulating enzymes such as lipoxygenase [15,16,17].

### 2.1. Lipid Peroxidation of Polyunsaturated Fatty Acids

DHA is a polyunsaturated fatty acid (PUFA) that exhibits various biological and physiological activities in organs, especially the brain [18,19]. DHA and EPA are included in membrane phospholipids. DHA- and EPA-containing lipids induce a negative curvature strain and markedly reduce membrane bilayer thickness, leading to an increase in membrane fluidity and permeability. DHA performs several biological and physiological activities in the body [18]. AD-associated pathologies include mitochondrial impairment, Aβ accumulation, neuroinflammation, neuronal loss, and impairment of adult hippocampal neurogenesis [20]. However, DHA is the most sensitive to intracellular RONS, which leads to the generation of bioactive products [21]. Thereby, oxidized DHA induces cell death in cultured cells [22]. DHA induces large aggregates and is involved in lipid peroxidation [23]. DHA and/or oxidized DHA induce misfolded proteins in cultured cells [24]. The modification of DHA oxidation levels in tissues and organs is essential to maintaining cellular fate, such as cell viability and cell death.

### 2.2. PUFAs and the Peroxidation Products in Neurodegenerative Diseases

In AD patients, DHA concentrations are reduced [25]. To date, DHA and EPA have been administered to treat major depression and AD [26]. ω-3 fatty acids are promising in both AD and PD [27]. The improvement in AD induced by polyunsaturated fatty acids is multifunctional. Among the PUFAs, DHA is the most potent anti-inflammatory fatty acid. D1, the bioactive DHA-derived lipid mediator neuroprotectin, has anti-neuroinflammatory and anti-apoptotic effects in neural cells [28]. On the other hand, DHA hydroperoxides induce the generation of ROS and cell death in human neuroblastoma SH-SY5Y cells [29]. In addition, DHA is a key molecule capable of binding α-synuclein and regulating its structure and function [30]. Interestingly, DHA promotes α-synuclein aggregation [31]. The structure and morphology of α-synuclein aggregation products are different from those in the presence of DHA. This report also shows that lipid oxidative products from DHA bind the methionine of α-synuclein [32]. These reports show that lipid peroxidation products can demonstrate both positive and negative regulation of protein aggregation processes.

The oxidative products from unsaturated fatty acids, such as DHA, EPA, and ARA, have bifunctional activities, which are both pro- and anti-inflammatory [12,33,34]. Highly reactive chemicals, such as acrolein, HNE, and MDA, are detected under pathological conditions [10,35]. These products are detected in non-apoptotic cells under various pathological conditions, including ischemia-reperfusion [36]. These products have also been identified in neurodegenerative diseases, such as AD, ALS, and PD [37]. Moreover, the products demonstrate their toxic properties in these neurodegenerative diseases [38,39]; lipid peroxidation triggers various neurodegenerative diseases [40,41]. As described above, ferroptosis via lipid peroxidation is a potent target for neurodegeneration therapy. Lipid–lipid aggregation of α-synuclein has been identified as a pathological characteristic in patients with PD [41]. α-synuclein exists as multiple types of oligomer, which are designated as type A* and type B*. Type-B* (toxic) oligomers disrupt lipid bilayers more strongly than type A* (non-toxic) oligomers [42]. Amyloid-like aggregate formation is regulated by various factors, including lipids [43]. One of the therapeutic targets of neuropathy is the interaction between lipids and aggregated proteins [44]. However, the mechanisms underlying the toxic effects of lipid peroxidation products and their induction of cell death are still unclear.

## 3. Cell Death Induced by Lipid Peroxidation

### 3.1. Ferroptosis

**Figure 2:**
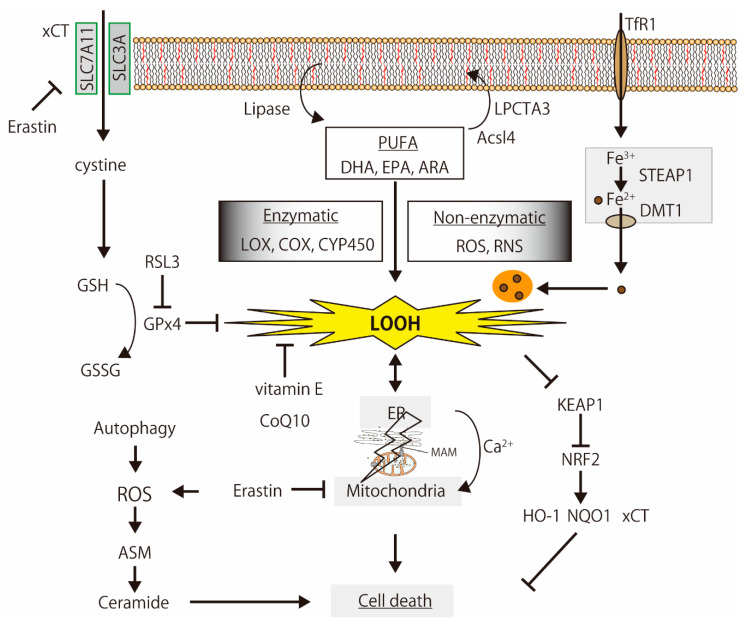
The simplified mechanisms of lipid peroxidation-induced cell death. PUFAs are oxidized by enzymatic and non-enzymatic reactions, and the products induce organelle dysfunction, iron-metabolism disturbance, Ca^2+^-signaling alternation, and cell death, which are regulated by the glutathione (GSH) synthesis signal, antioxidants, and oxidative stress response. The illustration was adapted from Iuchi (2021).

Ferroptosis is non-apoptotic cell death via lipid peroxidation; it is iron-dependent and causes glutathione depletion [45,46] (Figure 2). Conrad et al. reviewed the functions and regulation of lipid peroxidation, ferroptosis, and the antioxidant network in diverse species [47]. Ferroptosis is involved in various diseases, such as neurodegenerative disorders and kidney, liver, and cardiovascular diseases [48,49]. To date, several ferroptosis-related enzymes, including acyl-CoA synthetase long-chain family member 4 (ACSL4), lysophosphatidylcholine acyltransferase 3 (LPCAT3), arachidonate lipoxygenases (ALOXs, especially ALOX15), cystine glutamate antiporter (xCT), GPx4, and nuclear factor erythroid 2 like 2 (NFE2L2, also known as NRF2), are involved in the ferroptosis execution pathway [50]. Lipoxygenases, including cytochrome P450 (CYP450), metabolize (oxidizes) unsaturated fatty acids, and lipid peroxides from unsaturated fatty acids play critical roles in ferroptosis. The peroxidation of PUFAs by lipoxygenases drives ferroptosis [51]. Acetaminophen (APAP)-induced ferroptosis in mouse liver is prevented by genetic inhibition of ACSL4 or lipid peroxidation inhibitor (vitamin E) supplementation [52]. These ferrotosis-related enzymes and antioxidants regulate neuronal cell function [53].

Ferroptosis is involved in various diseases, such as neurodegeneration and kidney, liver, and cardiovascular diseases [47,48]. The therapy for neurodegenerative diseases is targeted at ferroptosis [54,55]. Masaldan et al. reviewed the relationship between neurodegeneration and ferroptosis [56]. In addition to ferroptosis, it is well-known that autophagy is related to neurodegenerative diseases [57]. The degradation of misfolded proteins and the malfunctioning of organelles by autophagy is pivotal to ferroptosis processes [58]. Huntington’s disease (HD) is an autosomal, dominant and fatal neurodegenerative disorder. GPx4 activation, NRF2-mediated signaling pathways, and iron transportation play a critical role in both HD and ferroptosis [48,59]. Amyloid β causes severe metabolic reprogramming associated with mitochondrial dysfunction, a well-known consequence of oxytosis/ferroptosis [60,61]. Taken together, the intracellular accumulation of misfolded proteins is the main cause and/or result of lipid peroxidation-induced cell death via organelle dysfunction.

Acid sphingomyelinase (ASM), a key enzyme in sphingolipid metabolism, is activated during ferroptosis, leading to the accumulation of ceramide [62]. The levels of ceramide and lysophosphatidylcholine accumulate during ferroptosis [51]. Alterations in sphingolipids, including ceramide, are associated with neurodegenerative diseases [63]. During ferroptosis, the change in lipid content is a pivotal event.

### 3.2. Relationship between Lipid Peroxidation and Calcium Signaling Pathway

Several lipid peroxidation products interact with calcium channels in the plasma membrane, which play the roles of an agonist and an antagonist [13,64]. Calcium influx is a major mechanism underlying the response to reactive lipid peroxidation products [65]. Lipid peroxidation occurs early during ferroptosis induced by Erastin and RSL3, leading to membrane rupture, cytosolic calcium increase, and cell rounding [66]. The prevention of lipid peroxidation restores calcium dysregulation in human iPSC-derived neurons with the triplication of the *SNCA* gene [67]. ER and mitochondria are calcium-storing organelles that communicate with each other. The mitochondrial and ER proteins involved in primary cell death mechanisms in cancer are well-known [68]. Mitochondria-associated membranes (MAMs) are new targets for AD because they are associated with calcium homeostasis and lipid metabolism in AD [69]. Alterations in Ca^2+^ signaling are involved in neurodegenerative diseases [70]. The dysregulation of intracellular Ca^2+^ signaling by lipid peroxidation in neurodegenerative diseases may be the primary process underlying cell death signaling.

## 4. Alternation of Organelle Function Regarding Lipid Peroxidation and Protein Aggregation

**Figure 3:**
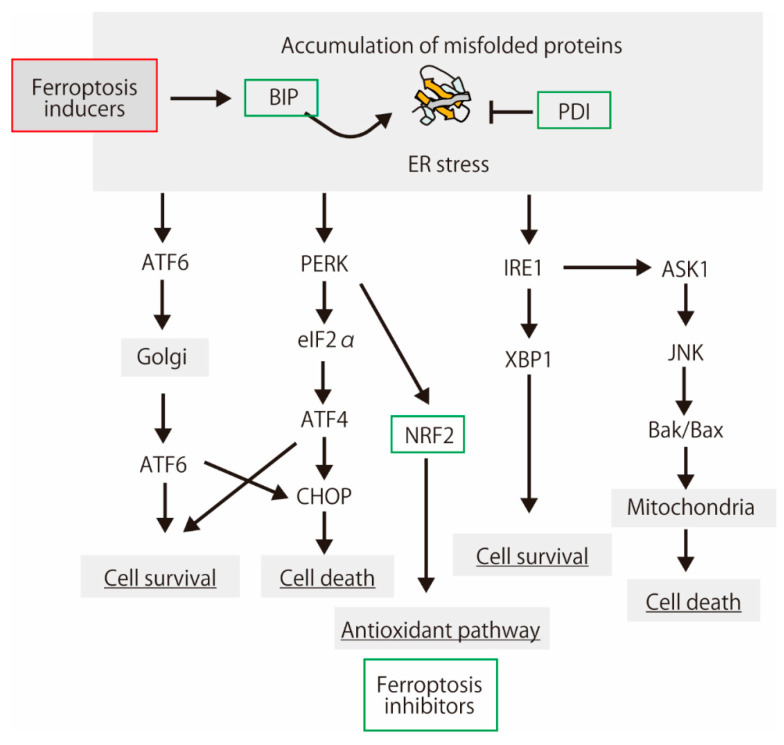
A schematic showing the protein aggregation, endoplasmic reticulum (ER) stress response, and cell fate. Protein aggregation is a main feature of the neurodegenerative diseases [88]. Some of the proteins induced by ER stress have ferroptosis-inhibiting activities.

**Figure 4:**
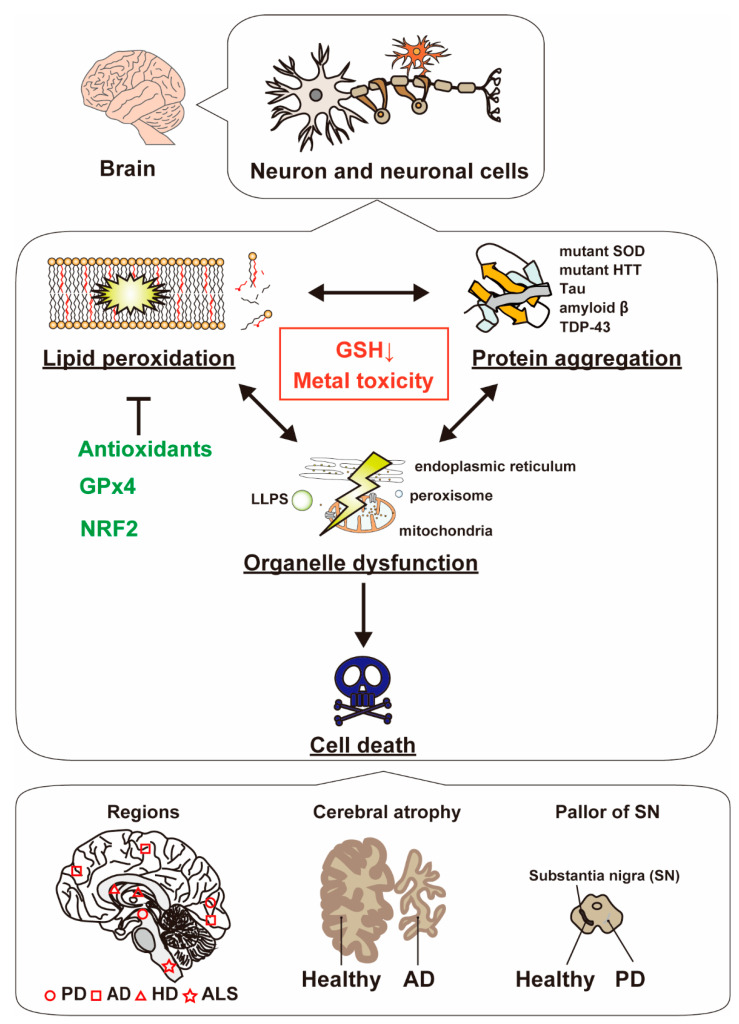
The molecular link between lipid peroxidation and protein aggregation in neurodegenerative diseases. Both lipid peroxidation and protein aggregation simultaneously occur in neuron and neuronal cells, leading to organelle dysfunction and cell death. ALS is a neurological disease characterized by progressive degeneration of nerve cells in the brain and spinal cord. Mutant superoxide dismutase 1 (SOD1) is expressed in a subgroup of familial ALS. Similarly, HD is caused by the huntingtin protein (HTT). The mutant proteins aggregate in the striatum of HD patients. In AD patients, tau fibrils and amyloid β aggregates are detected in the brain. Cerebral atrophy, which is the loss of cerebral brain cells, also occurs in AD patients. In PD, although α-synuclein is increased, the number and sizes of dopaminergic neurons are reduced in the substantia nigra. The bottom panel indicates major neurodegenerative diseases and their associated regions [120,121].

Organelles play important roles in lipid peroxidation-related protein aggregations, which are related to neurodegenerative diseases (Figure 3 and Figure 4). Organelle function is altered by both lipid peroxidation and misfolded protein accumulation of misfolded proteins. The mechanism of organelle dysfunction is as follows: highly reactive lipid peroxidation products increase the misfolding protein levels, as shown below.

### 4.1. Mitochondrial Dysfunction and Misfolded Proteins

Mitochondria are referred to as the powerhouse of cells, and they produce ATP via oxidative phosphorylation, which simultaneously releases reactive oxygen species such as superoxide anion radicals, and forms mitochondrial respiratory complexes. Under normal conditions, the radicals in mitochondria are efficiently scavenged by antioxidative systems (antioxidants and antioxidative enzymes in mitochondria). On the other hand, under stress conditions, mitochondrial function is impeded by reactive molecules such as RONS. Mitochondria are known as a source and target of lipid peroxidation [71]. Mitochondrial dynamics are altered in neurodegenerative diseases [72]. The administration of nordihydroguaiaretic acid (NDGA), an antioxidant, improves mitochondrial function and neuropathology in HD model mice [73]. In addition, as described below, antioxidants demonstrate neuroprotective activities through mitochondrial protection [74,75].

Several reports have shown the role of mitophagy in selected neurodegenerative diseases. Mitochondrial malfunction (dysfunction) is pivotal in neurodegenerative diseases [76]. PTEN-induced kinase 1 (PINK1) and E3 ubiquitin ligase Parkin play important roles in mitochondrial quality control. PINK1/Parkin is activated in mitochondrial removal (mitophagy) and regeneration [77].

Mitochondrial membrane phospholipids contain unsaturated fatty acids, which are essential for maintenance and function [78]. PUFAs, especially DHA, affect mitochondrial membrane phospholipid composition and mitochondrial function [79]. Cardiolipin, a tetra-acyl phospholipid, comprises 10–20% of the total mass of mitochondrial phospholipids, which is increased by supplementation with n-3 PUFA. Moreover, cardiolipin plays a key role in cell death signaling [80]. Cardiolipin is oxidized under oxidative stress, leading to mitochondrial dysfunction, including the inactivation of Complexes I, III, and IV of the respiratory chain [81]. Mitochondrial proteins aggregate under oxidative stress. For example, a GFP-tagged UCR-11, a complex III electron transport chain protein, is aggregated by aging or hypoxic stress [82]. Protein aggregates (ProteoStat-staining proteins) accumulate in breast cancer cell lines with dysfunctional mitochondria [83]. ProteoStat is a dye that can intercalate in the cross-spine of aggregated proteins. Cardiolipin plays a vital role in α-synuclein folding [84]. These reports suggest that oxidative stress induces the accumulation of mitochondrial lipid and protein aggregations.

### 4.2. Endoplasmic Reticulum Stress and Lipid Peroxidation

The endoplasmic reticulum (ER) is a key organelle involved in intracellular calcium storage and protein homeostasis (synthesis, degradation, and modification). When the ER-associated degradation system is unregulated, the misfolded proteins accumulate, leading to protein aggregation and ER stress [85]. The accumulation of misfolded proteins in the ER is enhanced during oxidative stress and results in ER stress, which, together, lead to the malfunction of cellular homeostasis [86]. Therefore, ER stress is closely related to various diseases, including neurodegenerative diseases, diabetes, metabolic syndromes, and cancer [87].

As shown below, the interaction between ER stress and ferroptosis has been reported. Dihydroartemisinin demonstrates a potent anticancer activity through ferroptosis, which is inhibited by the unfolded protein response involving the protein kinase R-like ER kinase (PERK)-upregulated activating transcription factor 4 (ATF4) and heat shock protein family A (Hsp70) member 5 (HSPA5, Bip) [89]. ATF4 induces angiogenesis in human glioma, which is diminished by ferroptosis inducers and GPx4 inhibitor [90]. Bip negatively regulates erastin-induced ferroptosis in human pancreatic ductal adenocarcinoma cells [91]. Erastin is a ferroptosis inducer; it inhibits xCT and voltage-dependent anion channels (VDACs), leading to lipid peroxidation [45,92]. Lipid hydroperoxides accumulate predominantly in the ER compartment [93]. Thus, ER stress and lipid peroxidation are cross-linked [94]. However, ER stress is not involved in ferroptosis induction, at least in melanoma cells [95]. Feng and Stockwell supposed that ER stress is simply a consequence of glutathione depletion during ferroptosis, and it does not contribute to the lethal mechanism [96]. Proteins in the endoplasmic reticulum membranes are modified by free radicals in both lipid-mediated and lipid-peroxidation-independent manners in vitro [97]. Therefore, ER proteins may be targets for lipid radicals from the plasma membrane.

The endoplasmic reticulum-resident peroxidases, such as GPx7 and GPx8, play pivotal roles during oxidative protein folding. GPx7 and GPx8 improve the ER antioxidative capacity of rat β-cells [98]. Especially, GPx7 forms complexes preferentially with protein disulfide isomerase (PDI) family proteins, such as PDI and P5 (PDIA6), in H_2_O_2_-treated cells [99]. The peroxiredoxin family of antioxidant enzymes, peroxiredoxin 4 (Prx4), preferentially recognizes two PDI family proteins, P5 and ERp46, and regulates the oxidative protein folding [100]. Recently, it has been reported that PDI family member P4HB, a functionally uncharacterized protein NT5DC2, a member of the haloacid dehalogenase (HAD) superfamily, are referred to as ferroptosis modulators [101]. These ER proteins, including PDI families, may be activated by involved in lipid peroxidation-induced ER stress in ER.

In pathological tissues, ER and oxidative stresses occur simultaneously. UPR regulates oxidative-stress-response signaling [102]. PERK upregulates NRF2 phosphorylation and dissociation from Keap1 [103,104]. Oxidative stress, NRF2 activation, and ER stress occur in human and mouse AD models [105,106]. Lipid peroxidation induced by oxidative stress induces the accumulation of misfolded proteins. The precise protein structure in the ER is essential for cell function. Ferroptosis via lipid peroxidation was inhibited by a protein synthesis inhibitor (cycloheximide) as well as deferoxamine (DFO), antioxidant trolox, and U0126, the MEK inhibitor [45]. ER stress also activates NRF2 in zebrafish [107]. The link between oxidative stress and ER stress is evolutionarily conserved among vertebrates. These reports suggest that the cellular response to misfolded proteins is partially concomitant with the response to oxidative stress, including lipid peroxidation.

### 4.3. Other Organelles

Fatty acids are metabolized in the peroxisomes, ER, and mitochondria. Peroxisomes, together with the ER, play a role in DHA synthesis. The degradation of oxidized fatty acids occurs in peroxisomes and mitochondria. The β-oxidation of short-, medium-, and long-chain fatty acids predominantly occurs in the mitochondria under physiological conditions. Peroxisomal dysfunction is related to neurodegenerative diseases and brain aging [108,109]. Perturbation of fatty acid composition in the brain is one of the triggers of neurodegenerative disease. Recently, peroxisome has been regarded as a new player in ferroptosis [110]. Ferroptosis is regulated by peroxisomal fatty acyl-CoA reductase 1, which catalyzes the formation of fatty alcohols via a reduction in saturated fatty acids [111]. Thus, saturated fatty acids, as well as unsaturated fatty acids, in peroxisomes may be pivotal factors.

α-synuclein, PD-related protein, and pre-formed fibrils (PFFs) are toxic to functional lysosomes in vitro [112]. Chloroquine and bafilomycin A1, which are lysosomal inhibitors, E-64D, which is a lysosomal cysteine protease inhibitor, and heparin inhibited the cytotoxicity of PFFs. MDA- or HNE-modified proteins are resistant to proteolytic degradation in lysosomal proteases [113]. Therefore, it seems that the highly reactive products of lipid peroxidation induce intracellular misfold proteins, which is concomitant with the perturbation of protein homeostasis in various organelles, including mitochondria, the ER, and peroxisomes. In addition, golgi apparatus also prevents ferroptotic cell death [114].

### 4.4. Liquid–Liquid Phase Separation

Liquid–liquid phase separation (LLPS) is regarded as a membrane-less organelle that plays critical roles in cellular functions. LLPS is the condensation of proteins, nucleic acids, or both in cells under stress conditions [115]. Cu^2+^, Fe^3+^, and liposomes accelerate the formation of α-synuclein oligomers and fibrillar species via LLPS [116]. The LLPS-induced formation of the Tau protein is essential for Tau aggregation, which is a pathogenic conformation [117,118]. Therefore, LLPS, composed of disordered proteins, is a novel target for neurodegenerative disease therapy [119]. LLPS-regulators such as metals overlap those of lipid-peroxidation-induced cell death, indicating that LLPS may be a new organelle that regulates and cleans the misfolded proteins induced by lipid peroxidation. Although it has not been reported that LLPS is detected during ferroptosis, LLPS may regulate the cell death signaling of ferroptosis. Therefore, further research is needed to prove this.

## 5. Manipulation of Neurodegeneration by Anti-Oxidative Chemicals and Enzymes

Patients with neurodegenerative diseases have abnormally high copper concentrations in their plasma and cerebrospinal fluid (CSF) [122]. For the clinical diagnosis of PD, the protein biomarkers in CSF were investigated [123]. The oxidized lipids produced during non-apoptotic cell death may form one of the PD candidates. Lipid peroxidation is associated with various oxidative stress-related diseases, including cardiovascular diseases, neurodegenerative diseases, and cancer [124,125,126]. Oxidative stress is a positive regulator of neurodegenerative diseases [127]. Therefore, various antioxidants have been used for therapy for neurodegenerative diseases [74]. Antioxidants (radical scavengers), such as coenzyme Q10 (CoQ10), edaravone, and α-tocopherol, inhibit the lipid peroxidation of PUFAs in vitro. The prevention of lipid peroxidation by these antioxidants is promising for treating oxidative stress-related diseases, such as neurodegenerative diseases [128,129,130,131]. These antioxidants are regarded as ferroptosis inhibitors. Thus, neuroprotection by antioxidants may be responsible for the elimination of lipid peroxidation followed by ferroptosis.

### 5.1. Anti-Oxidants and Electron Transfer Components

An antioxidant tripeptide, glutathione, is a direct scavenger of oxygen species and it plays a role as a co-factor for glutathione peroxidases. Glutathione is synthesized from glutamate, glycine, and cysteine. Cysteine is imported into cells directly or in its oxidized form, cystine, via the cystine/glutamate antiporter system [132], which consists of two subunits: the light chain subunit Solute carrier family 7 member 11 (SLC7A11, also known as xCT) and heavy chain subunit SLC3A2 (also known as CD98 or 4F2). Erastin inhibits cysteine uptake via xCT, induces ferroptosis, and triggers endoplasmic reticulum (ER) stress [133]. Oncogenic KRAS protects 3T3 fibroblasts from oxidative stress by enhancing intracellular glutathione levels through the transcriptional upregulation of xCT [134]. During ER stress, ER glutathione transport is activated and regulated by ER oxidoreductin 1 and BIP [135]. Thus, ferroptosis-induced ER stress may be associated with the inhibition of glutathione transport.

CoQ10 is a lipid-soluble antioxidant that has been clinically used in the treatment of various human disorders, such as metabolic syndrome, type 2 diabetes, cardiovascular diseases, and neurodegenerative diseases [136] As described above, CoQ10 inhibits ferroptosis, and there have been various reports on neurodegenerative diseases [75,137,138].

Vitamin E (alpha-tocopherol) is also a hydrophobic antioxidant that prevents the free-radical chain reaction of unsaturated lipids as a result of scavenging lipid radicals. Vitamin E in brain cells plays an important role in protecting cerebellar functions from oxidative damage [139]. Although the beneficial effects of vitamin E in vitro and in animal models have been reported, the outcomes of randomized clinical trials of vitamin E do not fully corroborate the effects [16]. The physical and chemical properties of vitamin E are altered by the experimental conditions [140]. An in vitro study showed that vitamin E shows potent ferroptosis-inhibitory activity in culture cells [141]. The mechanisms underlying the inhibition of ferroptosis by vitamin E are the direct scavenging of lipid radicals and the inhibition of 15-lipoxygenase via the reduction in its non-heme Fe^3+^ center to the inactive Fe^2+^ state by alpha-tocopherol hydroquinone [17]. In animal models, APAP-induced hepatotoxicity is a common cause of drug-induced acute liver failure, which is prevented by the genetic inhibition of the acyl-CoA synthetase long-chain family member 4 or α-tocopherol supplementation [52]. Low-density lipoprotein (LDL) is aggregated by a radical initiator, including CuSO_4_, which is inhibited by vitamin E [142]. Several antioxidants, such as NAC, vitamin C, and curcumin, inhibit protein aggregation in vitro, but vitamin E does not [143]. These reports suggest that the inhibitory effects of vitamins on protein aggregation are dependent on the elimination of lipid peroxidation, but not the direct binding to proteins.

### 5.2. Metal Ions

Metal ions are critically involved in the pathogenesis of major neurological diseases [144,145]. α-synuclein oligomer-induced ROS production is entirely dependent on the presence of free metal ions, which are blocked metal chelator metals (desferrioxamine, iron chelator, D-penicillamine—copper chelator, and clioquinol (CLQ)—highly lipophilic copper and zinc chelator with moderate affinity for iron-binding). These chelators prevent oligomer-induced neuronal death [146]. Iron is an essential metal involved in various intracellular processes, including oxidation-reduction reactions, DNA synthesis and repair, and other cellular processes. In general, the disturbance of intracellular iron homeostasis induces reactive oxygen and nitrogen species, leading to cell death via lipid peroxidation [147]. The brain is one of the organs in which iron accumulates with increasing age. An increase in iron may trigger ferroptosis. Thus, aggregated proteins may be produced during non-apoptotic cell death processes.

Metals, such as Cu^2+^ and Fe^3+^, regulate cellular functions and protein malfunction. Cu^2+^ and Fe^3+^ regulate the α-synuclein structure; Cu^2+^ induces thin long network-like fibrils with the wild-type α-synuclein and an amorphous aggregation of the α-synuclein mutants (A30P, A53T, and E46K) with no fibrillar forms [148]. Fe^3+^ induces short and thick fibrils with both wild and mutant forms. Similarly, Cu^2+^ modulates the morphology and characteristics of amyloid β(1–42) aggregates [149]. This report shows that amyloid β aggregates formed in the presence of Cu^2+^ degrade H_2_O_2_ and generate hydroxyl radical. Hydroxyl radical is the most highly reactive oxygen species, and it attacks unsaturated fatty acids. This indicates that metals are essential for the protein aggregation via the lipid peroxidation by hydroxyl radical. Interestingly, a novel chemical compound with free ladical scavenging activity is able to modify the aggregation of both metal-free amyloid β and metal−amyloid β [150]. Sphingosine, a 18-carbon amino alcohol with an unsaturated hydrocarbon chain, binds to amyloid β and metal, and alter the aggregation of both metal-free amyloid β and metal–amyloid β [151]. Zinc homeostasis is associated with the pathogenesis of neurological disorders, including PD, AD, and ASL [152]. For example, Zrt-like and Irt-like protein family members (ZIP) are predominantly expressed in the hippocampus and regulate neurodegeneration [153,154]. ZIP7 controls zinc transport from endoplasmic reticulum (ER) to cytosol [155]. Zinc protects against spinal cord injury-induced oxidative stress by activating nuclear factor NRF2 [156]. As in previous reports, zinc is also a confounding modulator of neurological function.

Therefore, various metals and bioactive lipids cooperatively modulate the protein biological properties in neurogenerative diseases. The oligomer formation of proteins is regulated by lipids and metals [157,158]. An excess number of metals may directly induce lipid peroxidation and protein oxidation followed by neurodegeneration, and the modulation of levels of metals may indirectly have a protective effect against lipid peroxidation via NRF2 activation.

### 5.3. Nuclear Factor (Esrythroid-Derived 2)-Like 2 (NRF2)

Nuclear factor (erythroid-derived 2)-like 2 (NRF2) is a transcription factor, which upregulates Phase II metabolism and cytoprotective genes, such as homo oxygenase-1 (HO-1), NADPH quinone dehydrogenase 1 (NQO1), glutamate-cysteine ligase modifier subunit (GCLM), and selenoproteins of the glutathione peroxidase (GPX) family. Thus, NRF2 is associated with autophagy, inflammation, apoptosis, mitochondrial biogenesis, stem cell function, and neurodegeneration [159]. Chen et al. reported NRF2-mediated neuroprotection in the mouse model of PD [160]. Nuclear NRF2 expression is increased in substantia nigra neurons from patients with PD [161]. This report shows that NRF2 in hippocampal neuronal nuclei decreases in AD, whereas nuclear NRF2 is predominant in the control. These reports suggest that the expression and localization of NRF2 are altered in neurodegenerative diseases, which are associated with oxidative stress. In addition, the endogenous antioxidant response pathway involved in the activation of NRF2 is associated with cancer cell death via lipid peroxidation. Shin et al. showed the mechanism by which p62 expression and the activation of the NRF2–ARE system are involved in the resistance to GPX4 inhibitor-induced ferroptosis in head and neck cancers [162]. NRF2 activation inhibits ferroptosis in hepatocellular carcinoma (HCC) cells [163]. Both ferroptosis and lipid peroxidation are mitigated by NRF2 activation [164]. NRF2 is not only activated in oxidative stress signaling, but also in ER stress [104]. Thus, NRF2 plays a pivotal role in the elimination of lipid peroxidation and protein aggregation.

The link between NRF2 function and neurodegenerative diseases has been recently demonstrated [165,166]. The expression of NRF2 is regulated by Kelch-like ECH-associated protein 1 (KEAP1), which is an adaptor subunit of Cullin 3-based E3 ubiquitin ligase. RONS-induced lipid peroxidative products, including 15-deoxy-D^12,14^-prostaglandin J_2_, 9-nitro-octadecenoic acid, and 4-HNE, bind KEAP1 cysteine residues, leading to the inactivation of KEAP1 and the upregulation of intracellular NRF2 [167]. ARA DHA, but not EPA, increased the intracellular 4-hydroxy hexenal (4-HHE), an end-product of n-3 PUFA peroxidation and the activation of NRF2 [34].

### 5.4. Glutathione Peroxidase 4 (GPx4)

As described above, glutathione peroxidase 4 (GPx4 or PHGPx) is a selenoprotein glutathione peroxidase, which is mainly localized in the nucleoplasm and mitochondria. GPx4 directly detoxifies lipid hydroperoxides in the membrane, thereby preventing lipid peroxidation (free-radical chain reaction). Thus, GPx4 plays an essential role in the brain because it maintains redox balance and mitochondrial function (Ca^2+^ homeostasis) and modulates neurogenesis [168]. GPx4 is exclusively expressed in neurons of the cerebellum, hippocampus, and hypothalamus, and it reduces lipid hydroperoxide [169]. During brain injury, GPx4 is also observed in reactive astrocytes. Moreover, both mRNA and protein expressions in GPx4 are diminished in multiple sclerosis and animal models of experimental autoimmune encephalomyelitis [170]. These suggest that GPx4 is the key regulator of neurodegeneration through the protection of lipid peroxide toxicity. Inactivation of GPx4 leads to neuronal cell death, which inhibits ferroptosis inhibitors [171,172]. Similarly, the glutathione synthesis-related proteins, including xCT and γ-glutamylcysteine ligase catalytic subunit (GCL), were reduced in experimental autoimmune encephalomyelitis. xCT and GCL are NRF2 target genes, and the expression and/or activation of NRF2 may be reduced.

### 5.5. Other Related Proteins

NADH:ubiquinone reductase (FSP1), which is also referred to as the apoptosis-inducing factor mitochondrion-associated 2 (AIFM2), is responsible for the suppression of ferroptosis. FSP1 is mediated by CoQ10. In addition, ACSL4 is a critical determinant of ferroptosis sensitivity [173]. ACSL4

## 6. Conclusions

This review summarizes data on the association between cell death via lipid peroxidation and protein aggregation in neurodegenerative diseases. The cellular and pathological phenomena characterizing lipid-peroxidation-related diseases are similar to those characterizing protein aggregation diseases. These phenomena have been separated because of the therapy described above. Subsequently, it will be necessary to fuse the two phenomena to facilitate the understanding of the onset mechanism of various diseases. We believe that the present review is useful for connecting the two important phenomena.
